# Enhanced spinal cord repair using bioengineered induced pluripotent stem cell-derived exosomes loaded with miRNA

**DOI:** 10.1186/s10020-024-00940-6

**Published:** 2024-10-01

**Authors:** Azar Abbas, Xiaosheng Huang, Aftab Ullah, Lishi Luo, Wenqun Xi, Yuanjiao Qiao, Kun Zeng

**Affiliations:** 1 Institute of Medicine, Shenzhen Institute of Advanced Technology, Chinese Academy of Sciences, 1068 Xueyuan Avenue, Nanshan District, Shenzhen, Guangdong 518055 P.R. China; 2 Department of Shenzhen Eye Hospital, Shenzhen Key Laboratory of Ophthalmology, Affiliated Hospital of Jinan University, No. 18, Zetian Road, Futian District, Shenzhen, Guangdong Province 518040 P.R. China; 3 School of Medicine, Huaqiao University, No. 269, Chenghua North Road, Fengze District, Quanzhou, Fujian 362021 P.R. China

**Keywords:** Spinal cord injury (SCI), Induced pluripotent stem cells-derived exosomes (iPSCs-Exo), miR-23b, miR-21-5p, miR-199b-5p, Etc

## Abstract

**Background:**

A spinal cord injury (SCI) can result in severe impairment and fatality as well as significant motor and sensory abnormalities. Exosomes produced from IPSCs have demonstrated therapeutic promise for accelerating spinal cord injury recovery, according to a recent study.

**Objective:**

This study aims to develop engineered IPSCs-derived exosomes (iPSCs-Exo) capable of targeting and supporting neurons, and to assess their therapeutic potential in accelerating recovery from spinal cord injury (SCI).

**Methods:**

iPSCs-Exo were characterized using Transmission Electron Microscopy (TEM), Nanoparticle Tracking Analysis (NTA), and western blot. To enhance neuronal targeting, iPSCs-Exo were bioengineered, and their uptake by neurons was visualized using PKH26 labeling and fluorescence microscopy. In vitro, the anti-inflammatory effects of miRNA-loaded engineered iPSCs-Exo were evaluated by exposing neurons to LPS and IFN-γ. In vivo, biodistribution of engineered iPSC-Exo was monitored using a vivo imaging system. The therapeutic efficacy of miRNA-loaded engineered iPSC-Exo in a SCI mouse model was assessed by Basso Mouse Scale (BMS) scores, H&E, and Luxol Fast Blue (LFB) staining.

**Results:**

The results showed that engineered iPSC-Exo loaded with miRNA promoted the spinal cord injure recovery. Thorough safety assessments using H&E staining on major organs revealed no evidence of systemic toxicity, with normal organ histology and biochemistry profiles following engineered iPSC-Exo administration.

**Conclusion:**

These results suggest that modified iPSC-derived exosomes loaded with miRNA have great potential as a cutting-edge therapeutic approach to improve spinal cord injury recovery. The observed negligible systemic toxicity further underscores their potential safety and efficacy in clinical applications.

**Supplementary Information:**

The online version contains supplementary material available at 10.1186/s10020-024-00940-6.

## Introduction

Spinal cord injury (SCI) is a devastating condition characterized by permanent damage to spinal nerves, leading to severe motor and sensory impairments (McDonald and Sadowsky 2002; Yepes 2007). The injury triggers a cascade of pathological events, including axonal damage, neuronal death, inflammation, and glial scarring, ultimately resulting in profound neurological and physical dysfunction (O’Shea et al. 2017; Goulão et al. 2019). Given the significant physical and psychological impact of SCI, elucidating its underlying mechanisms has been a major research focus. In a groundbreaking discovery, Yamanaka et al. introduced induced pluripotent stem cells (iPSCs) in 2006, which have since shown immense promise in treating neurological and cardiovascular diseases, including SCI (Takahashi and Yamanaka 2006). Subsequently, a series of researches have revealed that iPSCs have great potential value in the treatment of neurological diseases and cardiovascular diseases, especially in SCI (Hockemeyer and Jaenisch 2016; Kumar et al. 2018; Csobonyeiova et al. 2019).

Extracellular vesicles (EVs), including exosomes, microbubbles, and apoptotic bodies, are released by various cell types such as mesenchymal stem cells, endothelial cells, and tumor cells (Baradie et al. 2020). Exosomes, in particular, are approximately 30–150 nm in size and contain a diverse array of macromolecules. These nano-sized carriers facilitate intercellular communication by delivering their cargo to target cells, influencing local and systemic processes (Hong et al. 2014). Exosomes have emerged as versatile platforms with applications spanning disease biomarkers, therapeutic response predictors, and drug delivery vehicles (Hong et al. 2014; Batrakova and Kim 2015). Notably, exosomes offer a promising alternative to traditional carriers like liposomes and nanoparticles by evading rapid clearance and toxicity in the bloodstream (Kopac 2021). Therefore, exosomes have emerged as versatile drug delivery vehicles capable of carrying siRNA, miRNA mimics, miRNA inhibitors, mRNA, and proteins encoded by plasmid DNA (Mulcahy et al. 2014). These nanovesicles naturally transport various biomolecules, including mRNA, miRNAs, and proteins, to distant target cells through specific receptor interactions (Fuhrmann et al. 2015). This intrinsic targeting ability has positioned engineered exosomes as a focal point in gene therapy research. Studies have shown that the molecular cargo delivered by engineered exosomes can significantly impact cellular and tissue functions, including cell biology, tumor growth, and tissue repair (Qiu et al. 2018; Tian et al. 2014).

Several miRNAs have been identified for their neuroprotective effects in the context of inflammation and spinal cord injury recovery. Specifically, miR-23b has been shown to suppress pro-inflammatory cytokines and promote cell survival by modulating the inflammatory response (Xu et al. 2012). Similarly, miR-21-5p plays a crucial role in promoting neuronal survival and reducing apoptosis under stress conditions (Bhalala et al. 2012). Additionally, miR-199b-5p has been found to regulate neuroinflammatory pathways, contributing to reduced inflammation and enhanced neuronal regeneration after SCI (Wang et al. 2019). These miRNAs, when delivered via exosomes, hold significant potential for enhancing recovery in spinal cord injury.

Our study investigated the therapeutic potential of engineered iPSCs-Exo in vitro and in vivo. Results demonstrated that these engineered exosomes effectively promoted motor function recovery in an SCI mouse model. Furthermore, we observed decreased expression of miR-23b, miR-21-5p, and miR-199b-5p in LPS and IFN-γ stimulated neurons compared to controls. Notably, engineered iPSC-Exo loaded with miRNAs i.e. miR-23b, miR-21-5p, and miR-199b-5p exhibited neuroprotective effects against inflammation and enhanced spinal cord injury recovery. These findings highlight the potential of engineered iPSC-Exo as a novel therapeutic strategy for SCI.

## Materials and methods

### Mice

The BALB/c mice aged 4 to 8 weeks were purchased from Pasteur Institute (Tehran, Iran). All animal experiments and procedures have been approved by the ethics committee of Shenzhen Key Laboratory of Ophthalmology, Affiliated Hospital of Jinan University, Shenzhen, China. All the experiments were conducted according to the national specific ethical standards for biomedical research. The mice were kept in cages in ventilated rooms, providing plenty of food and water.

### iPSC culture and exosome preparation

The iPSCs used in this study were obtained from the Cell Resource Center, Peking Union Medical College. The iPSCs were cultured in exosome-free media for 48 h before being processed for exosome isolation. According to the previous studies (Théry et al. 2006; Gao et al. 2023), the engineering exosomes were separated by ultracentrifugation. Specifically, 0.4 mL of the iPSC cell culture supernatant was centrifuged at 300 x g for 10 min, transferred to a clean tube, and centrifuged at 2000 g for 20 min to remove cell debris. Subsequently, the supernatant was transferred to a clean test tube, centrifuged at 10,000 g for 30 min, and then vacuum filtered with a 0.22 μm filter (EMD, billica, MA). Furthermore, the supernatant was transferred to an ultracentrifuge tube (Beckman Coulter, Braille, CA) and centrifuged in an ultracentrifuge (Beckman Coulter optima TM Xe) 100,000 × g for 70–120 min. The resulting precipitate was washed once with cold, sterile phosphate buffered saline (PBS, pH 7.4) and resuspended in 0.3–0.6 mL PBS containing 1% DMSO. The protein concentration of the isolated exosomes was then measured using the Pierce™ BCA Protein Assay.

### Isolation and characterization of exosome

The Exosomes Isolation Reagent (Invitrogen, CA) was used to isolate exosomes from iPSCs. The iPSCs were first detached using Accutase (Innovative Cell Technologies, San Diego, CA) by incubating the cells at 37 °C for 5 min until they detached from the culture dish. The detached cells were collected by centrifugation at 300 g for 5 min and resuspended in PBS. After washing with PBS, the cells were resuspended in exosome-free media and cultured for 48 h. After that, the cells were centrifuged 3000 rpm for 15 min and the supernatants were collected. The supernatants were transferred to a fresh tube and exosome isolated reagent was added. The mixture was incubated overnight and then centrifuged at 15,000 rpm for 1 h to pellet exosomes. The pellet exosomes were resuspended with incomplete ESC medium or PBS. The morphology and the concentration particles of exosomes were detected by the transmission electron microscope (TEM) (FEI, USA) and nanoparticle tracking analysis (NTA) (ZetaView PMX110, Particle Matrix, Meerbusch, Germany), respectively.

### Exosome labeling and loading

Exosomes were observed using the fluorescent probe PKH67 (Invitrogen, Carlsbad, California, USA). A total of 100 µL of purified exosome suspension (with a protein concentration of 10 µg/mL) was incubated with 5 µL PKH67 at 37 °C for 15 min. The mixture was then centrifuged at 120,000× g for 90 min to remove unbound probes. After two rounds of washing and centrifugation, the labeled exosomes were suspended in PBS and subsequently used for cell research.

For miRNA loading, exosomes with a total protein concentration of 10 µg/mL were mixed with 400 nm Cy5-labeled miRNA in 1 mL PBS. The mixture was subjected to electrophoresis under the following conditions: 400 V, 50 µF, 30 ms pulse/2 s, for three cycles. After loading miRNA, the exosome samples were diluted 10 times with PBS and centrifuged at 110,000× g for 70 min to remove the unbound miRNA. The incorporation of miRNA into exosomes was determined by RT-PCR.

### Exosome uptake

iPSC-derived neuronal cells (3 × 10^5^) were seeded in a 3.5 cm glass bottom dish and incubated until 70% confluency was achieved. Then the cells were washed with PBS and incubated with cell culture medium containing 108 particles/mL PKH67-labeled foreign bodies. The fluorescence signals of PKH67 were recorded by confocal laser scanning fluorescence microscope (CLSM), and the images were processed by Zen software (CLSM; Zeiss lsm710, Oberkochen, Germany).

### RNA extraction and quantitative RT-PCR assay

Trizol reagent (Invitrogen, Carlsbad, California, USA) was used to extract total RNA from the samples. The M-MLV Reverse Transcriptase (RNase H-) kit (Invitrogen, CA) was employed to synthesize cDNA from the extracted RNA. This kit was chosen for its efficiency in producing high-quality cDNA by preserving the integrity of the RNA template, owing to its lack of RNase H activity. RT-qPCR was performed using SYBR Green PCR Master Mix (Takara, Kusatsu, Japan). GAPDH was used as the housekeeping gene for mRNA detection, and U6 snRNA was used as the housekeeping gene for miRNA detection. The relative expression levels were calculated using the 2^-ΔΔCT method. The primers used in this study were synthesized with sequences as shown in Table 1.

**Table 1:** Primer sequences used in the study

Gene/Target	Primer Type	Sequence (5’ to 3’)	Reference
GAPDH	Forward	GAAGGTGAAGGTCGGAGTC	Housekeeping gene for mRNA
GAPDH	Reverse	GAAGATGGTGATGGGATTTC	Housekeeping gene for mRNA
miR-21-5p	Forward	GCGGCGTAGCTTATCAGACTGATG	Specific for miR-21-5p detection
miR-21-5p	Reverse	GTGCAGGGTCCGAGGT	Specific for miR-21-5p detection
miR-23b	Forward	GGGTCCCTGAGAGTGTTTGA	Specific for miR-23-b detection
miR-23b	Reverse	GTGCAGGGTCCGAGGT	Specific for miR-23-b detection
U6 snRNA	Forward	CTCGCTTCGGCAGCACA	Housekeeping gene for miRNA
U6 snRNA	Reverse	AACGCTTCACGAATTTGCGT	Housekeeping gene for miRNA

### Western blot

Total proteins from tissues or cells were lysed in RIPA buffer and quantified by BCA analysis (Beyotime, Shanghai, China). The protein was analyzed by 10% twelve alkyl sulfate polyacrylamide gel (SDS-PAGE), and the gel was transferred to TBST sealed polyvinylidene fluoride (PVDF) membrane in 5% skim milk powder for 1 h. The PVDF membrane was incubated with the first antibody at 4 ℃ overnight: anti-TSG101, anti-CD63, anti-cytochrome C. β-actin was used as an internal control. All antibodies were purchased from (San Francisco, Santa Cruz, USA, 1:1000). The optical density of protein band was determined by Image J software, Inc.

### Rodent model of spinal cord injury

Mice were anesthetized with pentobarbital and underwent laminectomy at the T11-12 level. A weight-drop injury was induced using a 5 g rod dropped from a height of 5 cm. After the injury, the incision was closed, and the mice were placed in a controlled environment for recovery. The SCI mice were divided into two groups, receiving either PBS or iPSCs-Exo treatment. Recovery was assessed through behavioral and morphological evaluations. For the behavioral assessment motor function recovery was evaluated using the Basso Mouse Scale (BMS) at multiple time points (days 1, 7, 14, 21, and 28) by blinded observers. For morphological assessment, histological analysis was performed on the spinal cords using hematoxylin and eosin (H&E) staining to assess tissue architecture and Luxol fast blue (LFB) staining to evaluate myelin integrity and apoptosis. Significant improvements in tissue morphology and reduced apoptosis were observed in the iPSCs-Exo treated groups, especially those treated with miRNA-loaded exosomes.

### Flow cytometry assay

Cell apoptosis was measured by Annexin V-Fluos Apoptosis Detection Kit (Invitrogen, Catalog No. V13242). In detail, iPSC-derived neuronal cells in each treatment group were harvested and washed twice with PBS. Each precipitate was resuspended in 400 µL PBS at a concentration of 1 × 10^6^ cells/mL. Then, 100 µL of incubation buffer containing 2 µL of Annexin V-Fluos and 2 µL of propidium iodide from the kit was added to the cells in each experimental group. The samples were gently mixed and incubated for 15 min at room temperature in the dark. Following incubation, each group was analyzed by BD flow cytometry within 1 h.

### In vivo visualization of intravenously injected exosomes

To evaluate the biodistribution of miRNA (miR-23b, miR-21-5p and miR-199b-5p) engineered exosomes, 1 × 10^6^ cells were injected into the ventral side of 6-8-week-old female BALB/ c mice. 30 µg PKH67-labeled exosomes were injected into SCI model mice and healthy control mice as controls. 200 µL PBS + 5% glucose was injected into SCI model mice as background control. 5% glucose supports cell viability and reduces potential osmotic stress during intravenous injection. SCI model mice were anesthetized with 2.5% isoflurane at 3, 12, 24 and 48 h after administration, and small animal imaging system (Kodak, Rochester, New York, USA) was used. The fluorescence signal of each tissue sample was quantified in the freehand drawn target area.

### Tissue dissection and fluorescent microscopy

At the end of the experiment, the mice were euthanized by neck detachment. In addition to spinal cord tissues, tissues of interest (including lung, liver, kidney and spleen) were cut and cryopreserved in 5%, 10% and 14% sucrose, then frozen in OCT medium at -80 ℃ and sectioned with Leica CM1800 cryostat. The frozen tissue Sect. (8 μm thick) were fixed in acetone and their nuclei were stained with DAPI. To visualize the fluorescently labeled exosomes in the dissected tissues, we used the Cycling-3 cell imaging multimodal reader (Bio-Tek Instruments, Winooski, VT, USA). Fluorescent images were captured for further analysis.

### Statistical analysis

The mean ± SD represented data from three independent experiments. GraphPad Prism version 5.0 software (GraphPad Software, Inc.) was used for statistical analysis of all data. Unpaired Student t-test was used for comparison between two groups, and One-way ANOVA followed by tukey test was used for comparison within multiple groups. P value *< 0.05* indicated that the difference was statistically significant.

## Results

### Characterization of iPSCs-Exo

**Fig. 1:**
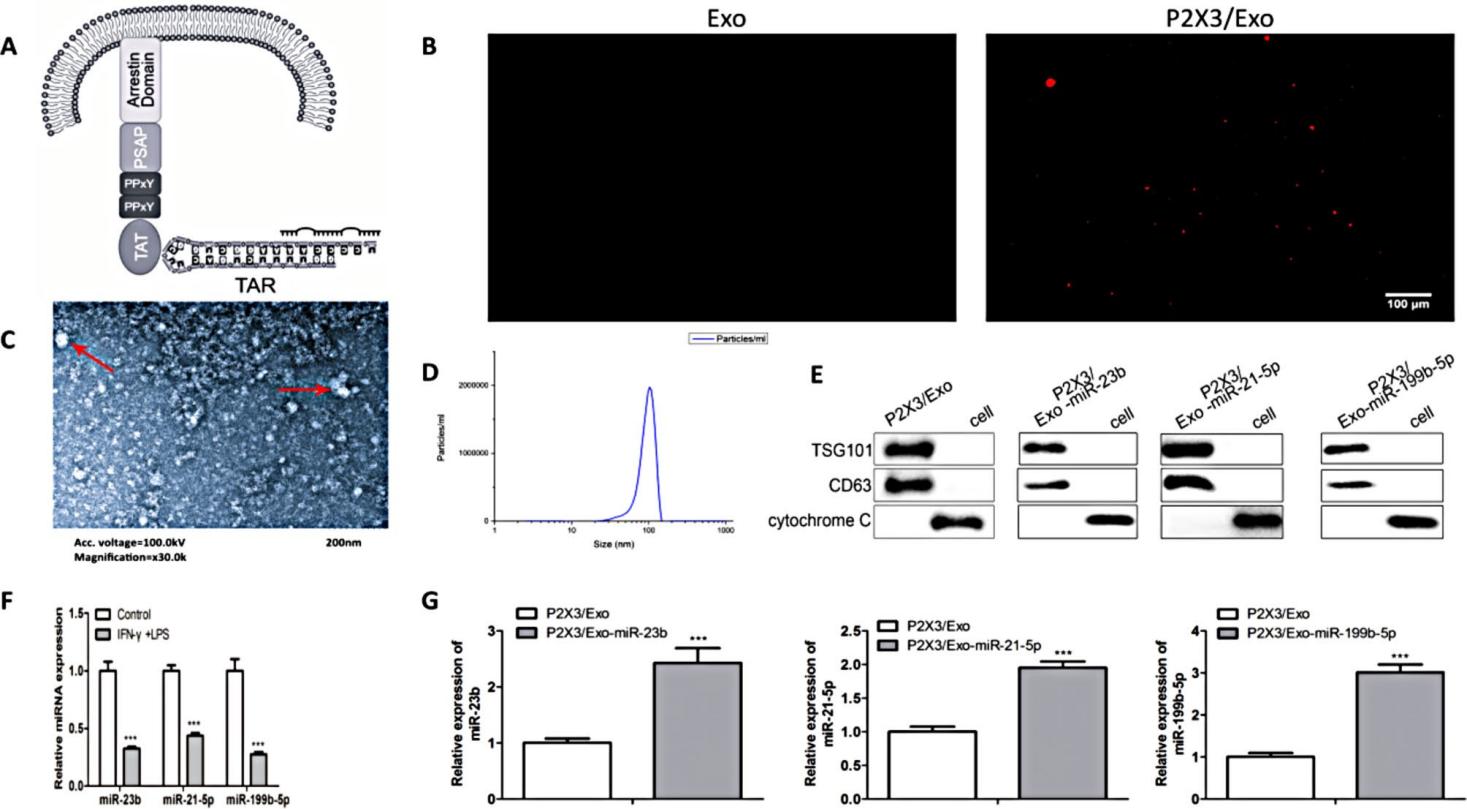
Characterization of iPSCs-Exo. (**A**) Diagram of plasmid construction. The sequence of events leading to loading of cargo miRNA into the iPSCs-Exo. (**B**) The fluorescence microscopy detects the location of exosomes. Scale bar: 100 μm. (**C**) transmission electron microscopy analysis of exosomes derived from iPSCs. (**D**) The particle size of iPSCs-Exo is measured by nanoparticle tracking analysis. (**E**) Western blot analysis of exosome surface markers CD63, TSG101 and Cytochrome C (*n* = 3). (**F**) The expression of miR-23b, miR-21-5p and miR-199-5p in the LPS and IFN-g induced neurons (*n* = 3). *P < 0.05, **P < 0.01, ***P < 0.001. (**G**) qRT-PCR analysis of miR-23b in P2 × 3/EXO and P2 × 3/EXO-miR-23b. qRT-PCR analysis of miR-21-5p in P2 × 3/EXO and P2 × 3/EXO-miR-21-5p. qRT-PCR analysis of miR-199-5p in P2 × 3/EXO and P2 × 3/EXO-miR-199-5p. (*n* = 3). * *P <* 0.05, **P < 0.01, ***P < 0.001

To establish engineered iPSCs-Exo, we constructed a plasmid to load cargo miRNA into the iPSCs-Exo. The miRNA was combined with the TAT peptide on the luminal C-terminus of the targeting protein (Fig. 1A). Fluorescence signals were used to select engineered iPSCs-Exo, and fluorescence microscopy detected these signals in the plasma membrane of exosomes (Fig. 1B).

P2 × 3 receptor, a purinergic receptor subtype, was incorporated into the engineered exosomes to enhance the targeting of iPSCs-Exo to neuronal cells. P2 × 3 is involved in sensory neuron signaling and plays a key role in neuroinflammatory pathways, making it a valuable target for enhancing exosome uptake in neuroinflammation models (Burnstock and Knight 2018).

The iPSCs-Exo were isolated from iPSCs, with TEM and NTA demonstrating their morphology and particle diameters. TEM and NTA analyses indicated that the exosomes were round vesicles with particles mainly ranging from 50 to 200 nm (Fig. 1C, D). Western blot results showed the expression of exosomal markers such as TSG101 and CD63, while cytochrome C was not expressed in the exosome group (Fig. 1E). These findings confirmed the successful isolation of iPSCs-Exo and iPSCs-P2 × 3/Exo.

To assess the anti-inflammatory effects of iPSCs-Exo, neuronal cells were treated with lipopolysaccharide (LPS) and interferon-gamma (IFN-gamma) to induce neuroinflammation. LPS and IFN-gamma are widely used to simulate the inflammatory environment observed in spinal cord injury (Rathinam and Fitzgerald 2016). qRT-PCR was then employed to measure miR-23b, miR-21-5p, and miR-199b-5p levels in this neuroinflammatory model, revealing that their expression was decreased compared to the control group (Fig. 1F). RT-qPCR further showed that miR-23b, miR-21-5p, and miR-199b-5p expression levels were significantly increased in the iPSCs-P2 × 3/Exo group compared to the iPSCs-Exo group (Fig. 1G), indicating enhanced delivery and uptake of miRNAs by neurons in the neuroinflammatory environment.

### Engineered iPSCs-Exo effectively enhanced targeted neurons in vitro

**Fig. 2:**
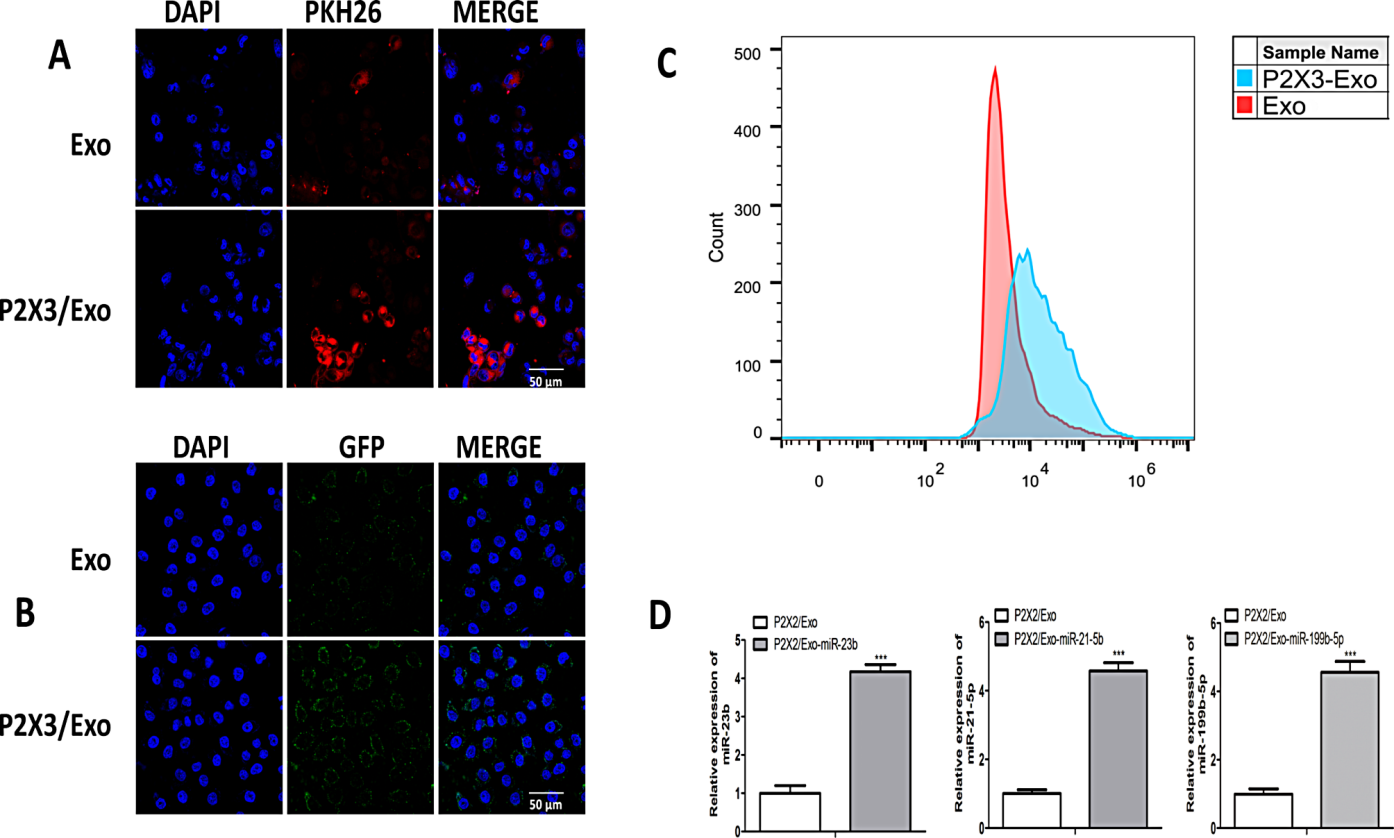
iPSCs-Exos localization in Neurons. (**A**) PKH26-labeled iPSCs-Exo or iPSCs-P2 × 3/Exo were co-cultured with neurons for 24 h. Representative immunofluorescence image showing PKH26-labeled (red) iPSCs-Exo or iPSCs-P2 × 3/Exo inside neurons, with the nuclei stained using DAPI (blue) (*n* = 3). Scale bar: 50 μm. (**B**) Confocal microscopy images showing GFP-positive neurons (green) co-cultured with iPSCs-Exo or iPSCs-P2 × 3/Exo for 24 h. DAPI (blue) was used for nuclear staining, and the merged images indicate the localization of the exosomes within the GFP-labeled neurons (*n* = 3). Scale bar: 50 μm. (**C**) Flow cytometry analysis demonstrating the percentage of neuronal cells that internalized PKH26-labeled iPSCs-Exo or iPSCs-P2 × 3/Exo after 24 h of co-culture. The enhanced uptake of iPSCs-P2 × 3/Exo is indicated by a higher fluorescence signal. (**D**) qRT-PCR analysis of miR-23b, miR-21-5p, and miR-199b-5p expression in neurons treated with iPSCs-P2 × 3/Exo loaded with miRNA compared to iPSCs-P2 × 3/Exo without miRNA. Data represent the mean ± SD (*n* = 3). *P < 0.05, **P < 0.01, ***P < 0.001

To assess neuronal uptake of exosomes, PKH26-labeled iPSCs-Exo and PKH26-labeled iPSCs-P2 × 3/Exo were co-cultured with neurons for 24 h. PKH26 is a fluorescent dye that labels the exosome membrane, allowing visualization of exosome internalization. Fluorescence microscopy revealed that both exosome types were internalized by neurons, with significantly greater uptake of PKH26-labeled iPSCs-P2 × 3/Exo compared to PKH26-labeled iPSCs-Exo (Fig. 2A). The enhanced uptake of iPSCs-P2 × 3/Exo is likely due to the presence of the P2 × 3 receptor, which facilitates more efficient targeting and internalization by neurons. Confocal microscopy confirmed these findings, demonstrating increased cytoplasmic localization and fluorescence intensity in GFP-positive neurons co-cultured with iPSCs-P2 × 3/Exo compared to those co-cultured with iPSCs-Exo (Fig. 2B). The term “GFP-positive” here refers to neurons that were transfected with GFP, allowing clear visualization of exosome uptake into neuronal cells. The GFP labels the neurons, while PKH26 labels the exosomes. Flow cytometry analysis further supported the enhanced neuronal uptake of iPSCs-P2 × 3/Exo (Fig. 2C). To evaluate the efficiency of miRNA delivery, engineered iPSCs-derived exosomes and those loaded with miRNA were co-cultured with neurons for 24 h. Subsequent qRT-PCR analysis demonstrated a significant increase in miR-23b, miR-21-5p, and miR-199b-5p expression in the neurons treated with miRNA-loaded iPSCs-P2 × 3/Exo compared to those treated with iPSCs-P2 × 3/Exo alone (*P <* 0.001) (Fig. 2D), confirming the successful delivery and accumulation of miRNAs within neurons.

### Engineered iPSC-derived exosomes protect neurons from inflammation

**Fig. 3:**
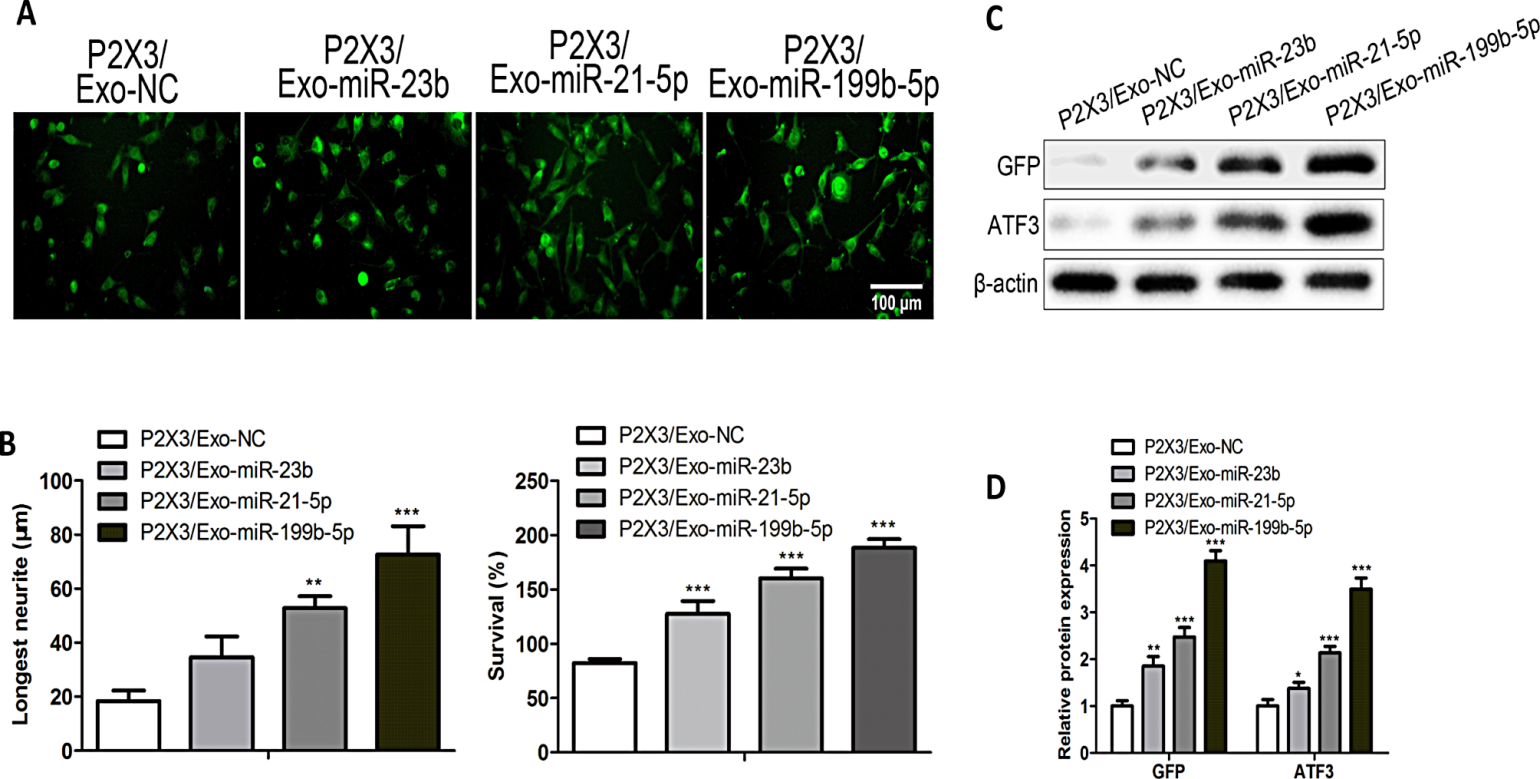
P2 × 3/Exo protected against inflammatory in neurons. (**A**) Representative immunofluorescence images of cultured neurons treated with P2 × 3/Exo-NC, P2 × 3/Exo-miR-23b, P2 × 3/Exo-miR-21-5p, or P2 × 3/Exo-miR- 199b-5p. Neurons were immunostained with anti-β-tubulin III (green) to label neuronal structure. While nuclei were initially stained with DAPI (blue), this signal is not visible due to the imaging settings (*n* = 3). Scale bars: 100 μm. (**B**) Quantification of neurite outgrowth and neuronal survival following treatment with P2 × 3/Exo-miR-23b, P2 × 3/Exo-miR-21-5p, and P2 × 3/Exo-miR-199b-5p. The length of the longest neurite was measured using ImageJ software, and the ratio of surviving neurons was assessed (*n* = 3). (**C**) Western blot analysis of GFP and ATF3 expression levels in cultured neurons after treatment with different exosomes. β-actin was used as a loading control (*n* = 3). Quantification of protein expression is shown relative to β-actin. *P < 0.05, **P < 0.01, ***P < 0.001

In order to detect the effects of P2 × 3 /EXO-miR-23b, P2 × 3 /EXO-miR-21-5p and P2 × 3 /EXO-miR-199b-5p on neurogenesis, we observe the therapeutic effect on neuronal cells. As shown in the figure S1, the NC mimics, miR-23b, miR-21-5p and miR-199b-5p mimics were transfected into LPS and IFN-g induced neurons. The results showed that the inflammation factors, including TNF-α and IL-6, were reduced in the miR-23b, miR-21-5p, and miR-199b-5p mimics transfected group. The anti- inflammation factors including IL-4 and IL-10 increased the miR-23b, miR-21-5p and miR-199b-5p mimics treatment group (Figure S1A) (*P <* 0.05). And we used flow cytometry to identify apoptotic neurons. After miR-23b, miR-21-5p and miR-199b-5p mimics treatment, the number of apoptotic cells decreased compared with the NC mimics group (Figure S1B) (*P <* 0.05). In addition, P2 × 3 /EXO-miR-23b, P2 × 3 /EXO-miR-21-5p and P2 × 3 /EXO-miR-199b-5p treatment increased neurite crosspoints. And the treatment of P2 × 3 /EXO-miR-199b-5p extraordinarily increased neurite crosspoints compared with the other groups (Fig. 3A) (*P* < 0.05). Compared with the P2 × 3/EXO-NC group, P2 × 3 /EXO-miR-23b, P2 × 3 /EXO-miR-21-5p and P2 × 3 /EXO-miR-199b-5p treatment increased β3-tubulin favorable longest neurite elongation. In addition, the treatment of P2 × 3 /EXO-miR-199b-5p extraordinarily increased the length of the longest neurite compared with the other groups. The number of survival neurons increased in the P2 × 3 /EXO-miR-23b, P2 × 3 /EXO-miR-21-5p and P2 × 3 /EXO-miR-199b-5p groups. In addition, The number of survival neurons is highest in the P2 × 3 /EXO-miR-199b-5p groups (Fig. 3B) (*P* < 0.05). The recent studies showed that GFP (Zariwala et al. 2012) and ATF3 (Gey and Schwab 2012) are the main proteins involved axonal growth. As shown in Fig. 3C, P2 × 3 /EXO-miR-23b, P2 × 3 /EXO-miR-21-5p and P2 × 3 /EXO-miR-199b-5p promoted the expression levels of GFP and ATF3. Neurite crosspoints and neurite length were visualized by fixing and staining these neurons with a β3-tubulin antibody and quantified by ImageJ (Fig. 3C) (*P* < 0.05). P2 × 3 /EXO-miR-23b, P2 × 3 /EXO-miR-21-5p and P2 × 3 /EXO-miR-199b-5p treatment increased neurite crosspoints, the length of longest neurite and the number of survival neurons.

### Engineered iPSCs-Exo localization in vivo

**Fig. 4:**
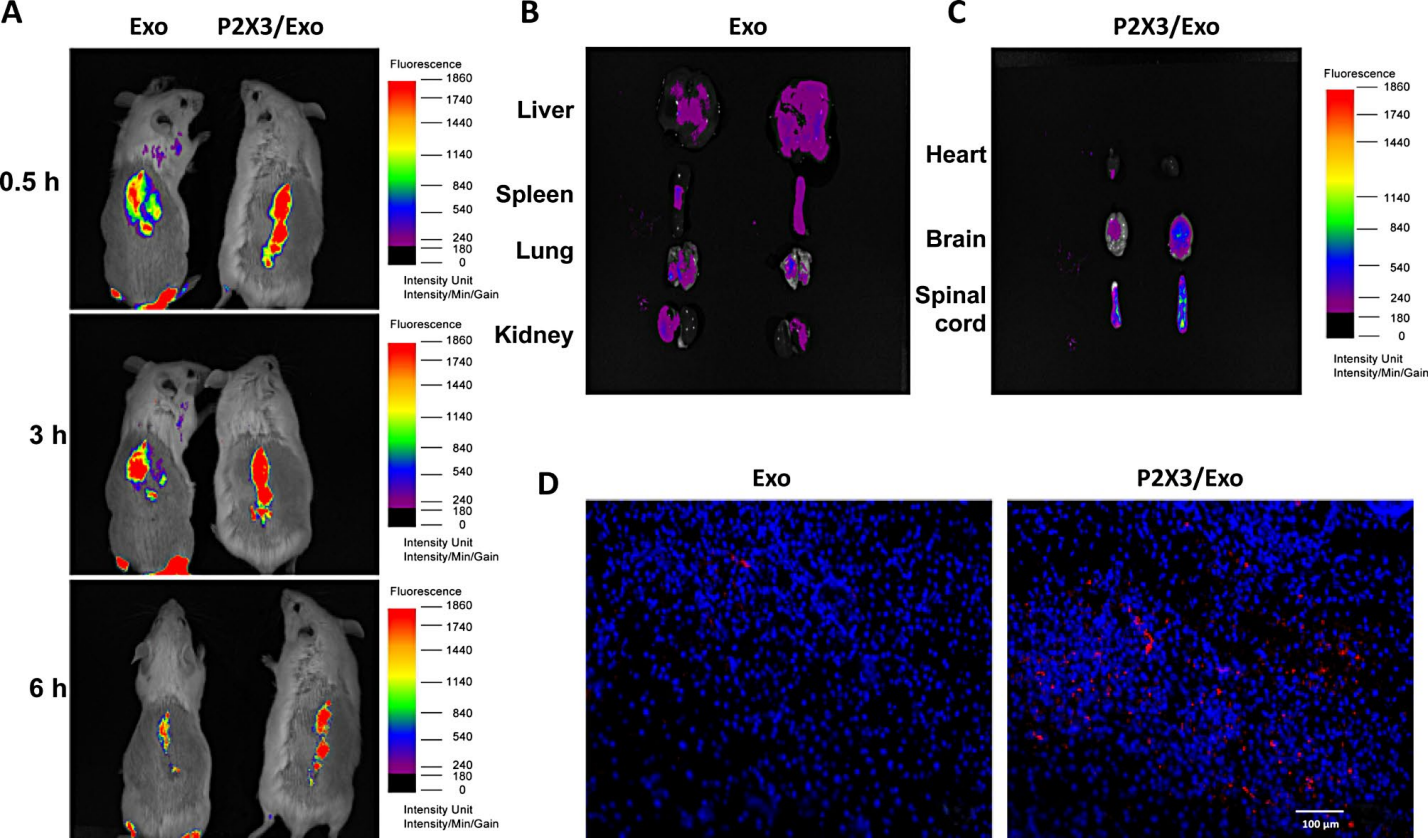
Engineered iPSCs-Exo localization in vivo (**A**) iPSCs-Exo and IPSCs-P2 × 3 /EXO were resuspended in PBS for intravenous injection. In vivo imaging system (IVIS) images were taken at different time points. (**B**) Imaging of fluorescence intensity in main organ. (*n* = 3) (**C**) The accumulation of fluorescence intensity in the spinal cord and brain after i.v. administration. (*n* = 3) (D) Fluorescent images showed IPSCs-P2 × 3 /EXO targeted neurons in spinal cord. Scale bars = 100 μm, *P < 0.05, **P < 0.01, ***P < 0.001

We established the spinal cord injury model and detect the biodistribution of iPSCs-Exo and IPSCs-P2 × 3/Exo in vivo by vivo imaging system. As shown in Fig. 4A, The fluorescence signals of IPSCs-P2 × 3 /EXO were significantly higher in the area of spinal cord injury (Fig. 4A) (*P* < 0.05). The results showed that fluorescence intensity in liver, spleen, lung and kidney was no significant difference in two groups (Fig. 4B) (*P* < 0.05). As shown in Fig. 4C, it was observed that compared with iPSCs-Exo, the IPSCs-P2 × 3 /EXO increased the accumulation of fluorescence intensity in the spinal cord and brain. In addition, the accumulation of fluorescence intensity was no significant difference in heart between two groups (Fig. 4C) (*P* < 0.05). Fluorescent images showed IPSCs-P2 × 3 /EXO targeted neurons in spinal cord compared with iPSCs-Exo (Fig. 4D) (*P* < 0.05).

### Engineered iPSCs-Exo regulated the spinal cord injury recovery

**Fig. 5:**
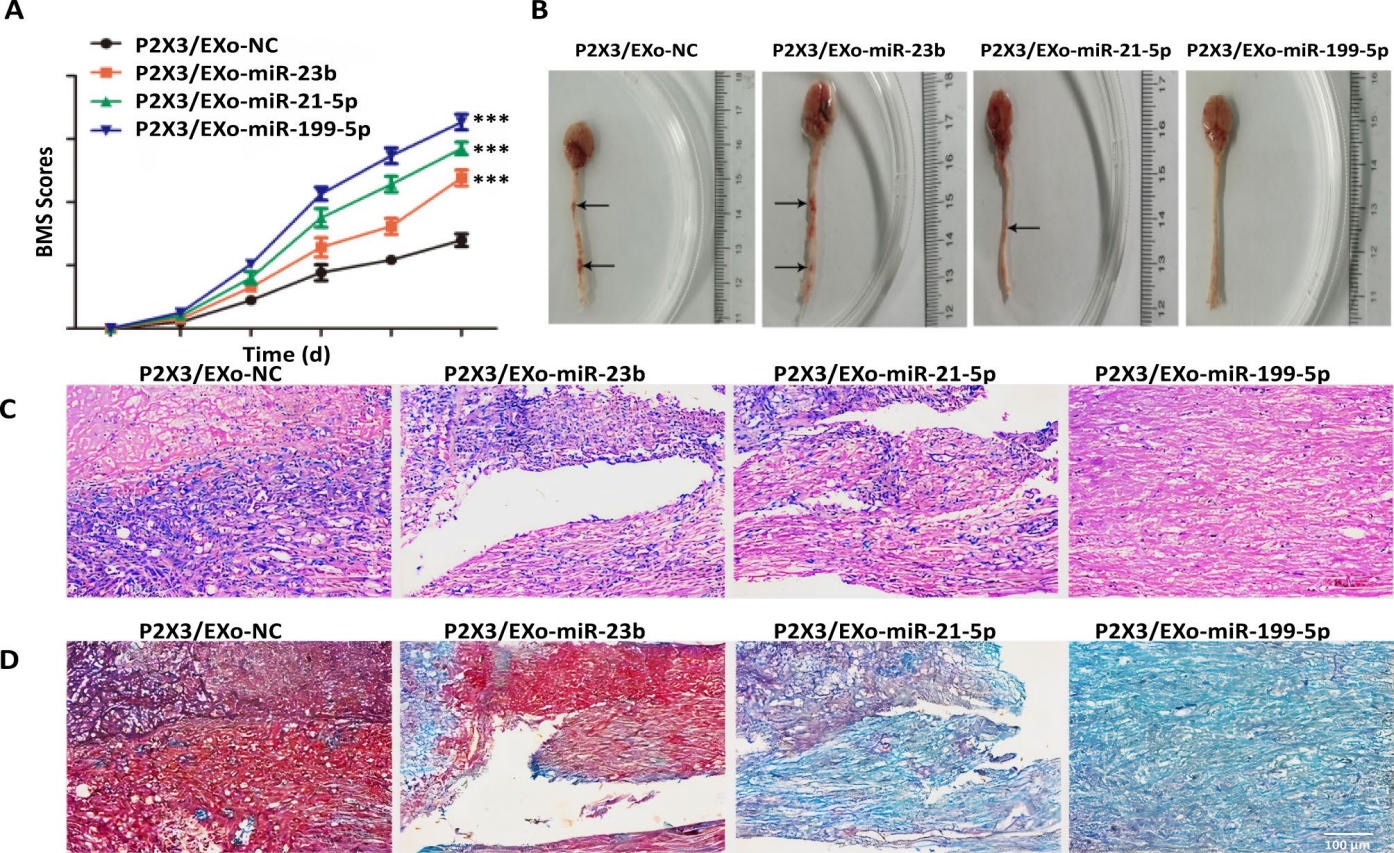
Engineered iPSCs-Exo regulated the spinal cord injure recovery (**A**) BMS was used to functionally grade the mice in the P2 × 3 /EXO-NC, P2 × 3 /Exo-miR-23b, P2 × 3/Exo-miR-21-5p and P2 × 3/Exo-miR-199b-5p groups (*n* = 3). (**B**) Gross morphology of spinal cord (*n* = 3). (**C**) Representative H&E staining images of the longitudinal sections of mice spinal cords (*n* = 3). (**D**) Representative LFB staining images of apoptotic neurons in the injured lesion areas of spinal cord. (*n* = 3) Scale bars = 100 μm, *P < 0.05, **P < 0.01, ***P < 0.001

To explore the recovery effects of engineered iPSCs-Exo in vivo, we established the spinal cord injury (SCI) mice model. The BMS scores were gradually improved with a time-dependent manner and increased in P2 × 3 /EXO-miR-23b, P2 × 3 /EXO-miR-21-5p and P2 × 3 /EXO-miR-199b-5p groups than the PBS group (Fig. 5A) (*P* < 0.05). In addition, the BMS scores was obviously higher in P2 × 3 /EXO-miR-199b-5p group than other groups (Fig. 5A) (*P* < 0.05). To better detect the recovery of injured spinal cords in SCI model mice, the mice were sacrificed and the spinal cords were observed. The results showed that the overall shape of the spinal cord has been significantly improved in P2 × 3 /EXO-miR-23b, P2 × 3 /EXO-miR-21-5p and P2 × 3 /EXO-miR-199b-5p groups when compared to the P2 × 3 /EXO-NC group. In the P2 × 3 /EXO-miR-199b-5p group, the overall shape of the spinal cord almost repaired. (Fig. 5B). Meanwhile, H&E staining was used to detect the longitudinal sections of mice spinal cords and observe the lesion area of SCI. The results showed that the inflammatory cell infiltration was reduced in P2 × 3 /EXO-miR-23b, P2 × 3 /EXO-miR-21-5p and P2 × 3 /EXO-miR-199b-5p groups. The inflammatory cell infiltration was significantly reduced in P2 × 3 /EXO-miR-199b-5p groups when compared with other groups (Fig. 5C). In addition, the recovery of spinal cord injure was better in engineered iPSCs-Exo and the recovery of spinal cord injure was better in P2 × 3 /EXO-miR-199b-5p groups compared with other groups (Fig. 5C). To observe the effects of engineered iPSCs-Exo on neuronal apoptosis, we used LFB staining to detect apoptotic neurons. After P2 × 3 /EXO-miR-23b, P2 × 3 /EXO-miR-21-5p and P2 × 3 /EXO-miR-199b-5p treatment, the number of apoptotic cells decreased compared with the P2 × 3 /EXO-NC group (Fig. 5D) (*P* < 0.05). These results suggested that Engineered iPSCs-Exo played essential role in the functional recovery after SCI.

### Toxicity and safety evaluation of Engineered iPSCs-Exo

**Fig. 6:**
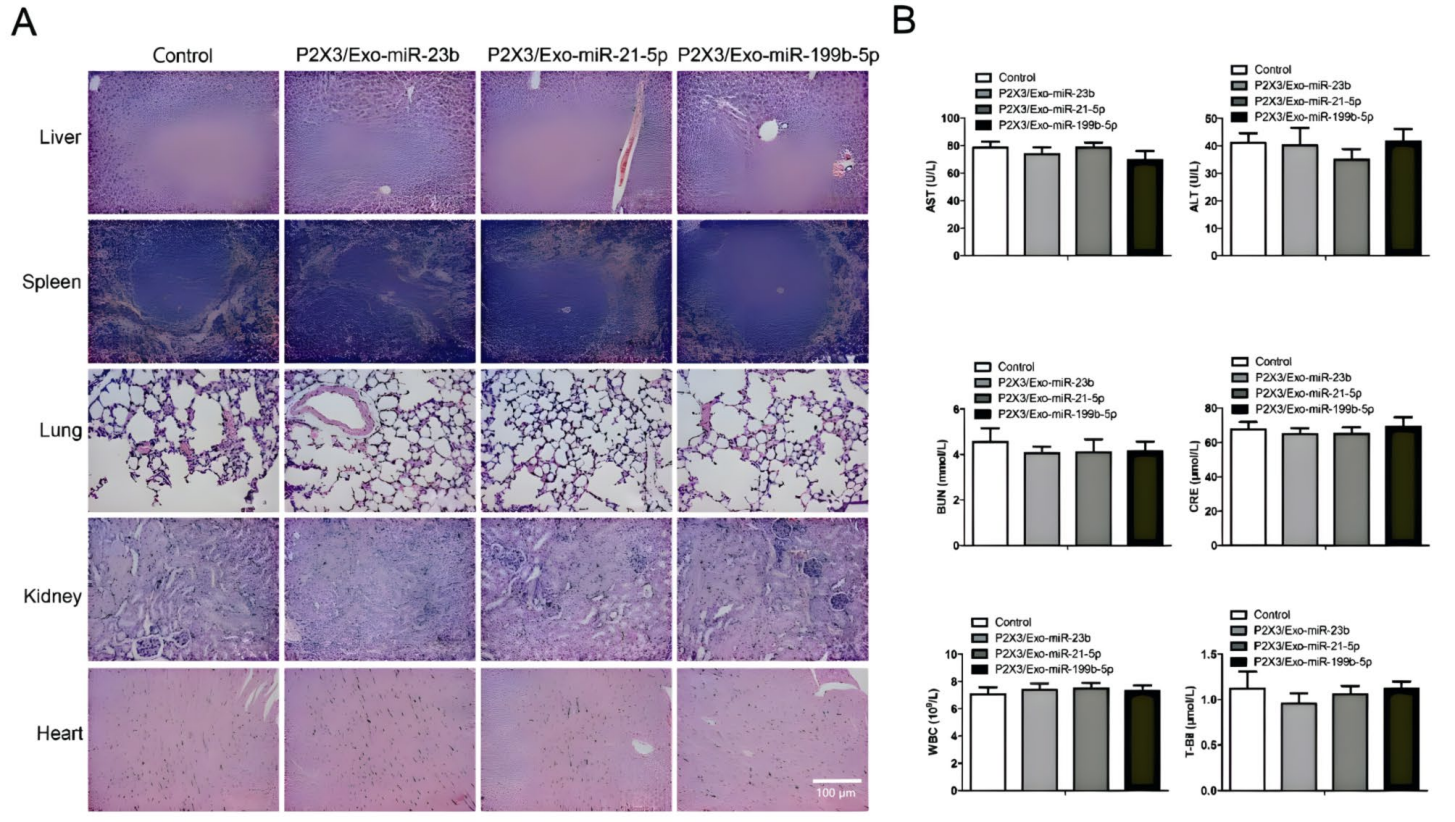
Safety evaluation of Engineered iPSCs-Exo. (**A**) H&E staining of major organ after treatment with engineered iPSCs-Exo and saline, scale bar = 100 μm. (**B**) Serum biochemistry and routine blood test results (AST, ALT, BUN, CRE, WBC and T-Bil) of mice receiving different treatments. (*n* = 3) Scale bars = 100 μm

In order to explore the Safety Engineered iPSCs-Exo in vivo, H&E staining was used to detect the sections of organs of interest. The results showed that there is no obvious pathological abnormality in main organs including liver, spleen, lung, kidney and heart (Fig. 6A). Meanwhile, the biochemistry parameters and blood routine have no significant difference between control group and Engineered iPSCs-Exo treatment group (Fig. 6B) (*P <* 0.05). During this study, it indicated negligible systemic toxicity.

## Discussion

Spinal cord injury (SCI) has long been a devastating condition with high rates of morbidity and mortality. Despite significant research efforts over the past decades, effective treatments for SCI remain limited (McDonald and Sadowsky 2002; Cofano et al. 2019; Siddall and Loeser 2001).

SCI remains a devastating condition with limited treatment options. Recent advancements in stem cell research have highlighted the potential of induced pluripotent stem cells (iPSCs) to revolutionize the treatment of various diseases, including SCI. Possessing the unique ability to self-renew and differentiate into multiple cell types, iPSCs offer a promising avenue for tissue regeneration and repair (Khazaei et al. 2014).

Previous studies have highlighted the critical role of paracrine mechanisms in the therapeutic efficacy of IPSCs (Katsuda et al. 2013; Hessvik and Llorente 2018). IPSCs-derived exosomes (iPSCs-Exo) have demonstrated comparable or superior therapeutic benefits across various diseases (Lou et al. 2017; Liu et al. 2019; Li et al. 2017; Guo et al. 2017; Huang et al. 2017). As extracellular vesicles (EVs), these nano-sized particles are secreted by mammalian cells and contain a diverse cargo of functional molecules, including proteins, DNA, and RNAs. EVs have been successfully employed as drug delivery vehicles for a range of biomolecules, such as siRNA, miRNA mimics, inhibitors, mRNA, and proteins (Raposo and Stoorvogel 2013; Simons and Raposo 2009; Zappulli et al. 2016). Exosomes, a subset of EVs, have emerged as promising therapeutic carriers due to their ability to deliver bioactive molecules to target tissues and cells. Encased in a protective lipid bilayer, exosomal cargo is shielded from degradation by proteases and nucleases. This unique characteristic, coupled with their potential for targeted delivery, makes exosomes an attractive platform for developing novel therapeutic strategies (Wood et al. 2011; Andaloussi et al. 2013; András and Toborek 2016; Xiong et al. 2017). Exosomes can take part in the therapeutic delivery as a new vehicle because of their promising ability to transfer bioactive molecules to target tissue and cells (Maas et al. 2017; Mäger et al. 2013). EVs are wrapped by a lipid bilayer membrane. Therefore, cargos which contain in the vesicles are protected against protease- or nuclease-mediated degradation. Recent studies have shown that we can use exosomes to deliver siRNAs, miRNA, miRNA inhibitors, mRNA and proteins to target tissue and cells (Alvarez-Erviti et al. 2011; Hung and Leonard 2016).

Recent studies have emphasized the pivotal role of miRNAs in regulating exosomal function. Given their ability to modulate entire signaling pathways, miRNAs are promising therapeutic targets for SCI (Chen et al. 2018). Previous research has demonstrated the anti-inflammatory effects of miR-146a-5p delivered via mesenchymal stem cell-derived exosomes (MSCs-EXO) in ischemic stroke (Zhang et al. 2021).

The therapeutic potential of iPSCs-Exo in promoting spinal cord injury recovery remains largely unexplored. While previous studies have demonstrated the modification of exosomal surfaces with chemicals, antibodies, and proteins to enhance targeted drug delivery, the development of efficient cargo loading strategies is essential. Our study focused on developing a system for loading miRNA into iPSCs-Exo and delivering it to target cells. To optimize miRNA loading, we leveraged the interaction between TAR RNA and TAT peptide, a strategy shown to enhance cargo incorporation into exosomes (Zou et al. 2019; Luo et al. 2019).

In this study, miR-23b, miR-21-5p, and miR-199b-5p were selected as therapeutic agents based on previous studies that demonstrated their important roles in regulating inflammation and promoting neuroprotection (Xu et al. 2012; Bhalala et al. 2012; Wang et al. 2019). These miRNAs have been shown to suppress pro-inflammatory cytokine production, enhance cell survival, and modulate neuroinflammatory pathways, making them ideal candidates for treating spinal cord injury (SCI).

In our SCI mouse model, we observed that miR-23b, miR-21-5p, and miR-199b-5p were significantly downregulated in LPS and IFN-γ-stimulated neurons, indicating their involvement in the neuroinflammatory processes post-SCI. To counteract this downregulation, we administered engineered iPSCs-derived exosomes (iPSCs-Exo) loaded with these therapeutic miRNAs. Our results demonstrated that these miRNA-loaded iPSCs-Exo significantly accelerated recovery post-SCI by promoting neuroprotection and attenuating neuronal inflammation.

These findings suggest that the therapeutic delivery of miR-23b, miR-21-5p, and miR-199b-5p via engineered exosomes can effectively modulate key inflammatory pathways, thereby reducing neuroinflammation and supporting neural recovery in SCI. This supports the potential of these miRNAs as key regulators in neuroinflammatory conditions and highlights the promise of exosome-based therapies for neuroprotection and recovery after SCI.

Furthermore, we observed a reduction in inflammatory cell infiltration and apoptosis following treatment. In vitro studies revealed enhanced neuronal targeting by engineered iPSCs-Exo. Notably, these exosomes accumulated in the injured spinal cord and brain.

Our findings demonstrate that engineered iPSCs-Exo loaded with miR-23b, miR-21-5p, and miR-199b-5p significantly enhance recovery from spinal cord injury (SCI) by attenuating inflammation in LPS and IFN-γ-stimulated neurons. These miRNAs play a critical role in mediating the therapeutic effects of engineered iPSCs-Exo in the SCI mouse model. Our results suggest that this innovative approach holds great promise as a potential treatment for SCI.

## Conclusion

Engineered iPSCs-Exo loaded with specific miRNAs offer a promising therapeutic strategy for spinal cord injury by effectively reducing inflammation and promoting recovery. While our findings highlight the potential of this approach, further in-depth investigations are necessary to fully elucidate the underlying mechanisms and optimize miRNA cargo. Long-term studies are crucial to assess the sustained therapeutic benefits and safety profile before clinical translation.

## Electronic supplementary material

Below is the link to the electronic supplementary material.

Supplementary Material 1

## Data Availability

No datasets were generated or analysed during the current study.

## Abbreviations

BMS: Basso Mouse Scale
DMSO: Dimethyl Sulfoxide
Exo: Exosome
GFP: Green Fluorescent Protein
H&E: Hematoxylin and Eosin
IFN-γ: Interferon Gamma
iPSCs: Induced Pluripotent Stem Cells
LFB: Luxol Fast Blue
LPS: Lipopolysaccharide
NTA: Nanoparticle Tracking Analysis
BS: Phosphate-Buffered Saline
RT-PCR: Reverse Transcription Polymerase Chain Reaction
TEM: Transmission Electron Microscopy
