# Supplementary material for: Enhanced spinal cord repair using bioengineered induced pluripotent stem cell-derived exosomes loaded with miRNA
Source: Mol Med. 2024 Oct 1;30:168. doi: 10.1186/s10020-024-00940-6 (PMC11446086; doi:10.1186/s10020-024-00940-6)
Supplement: Supplementary file 1 — Supplementary Material 1 [file 10020_2024_940_MOESM1_ESM.docx]

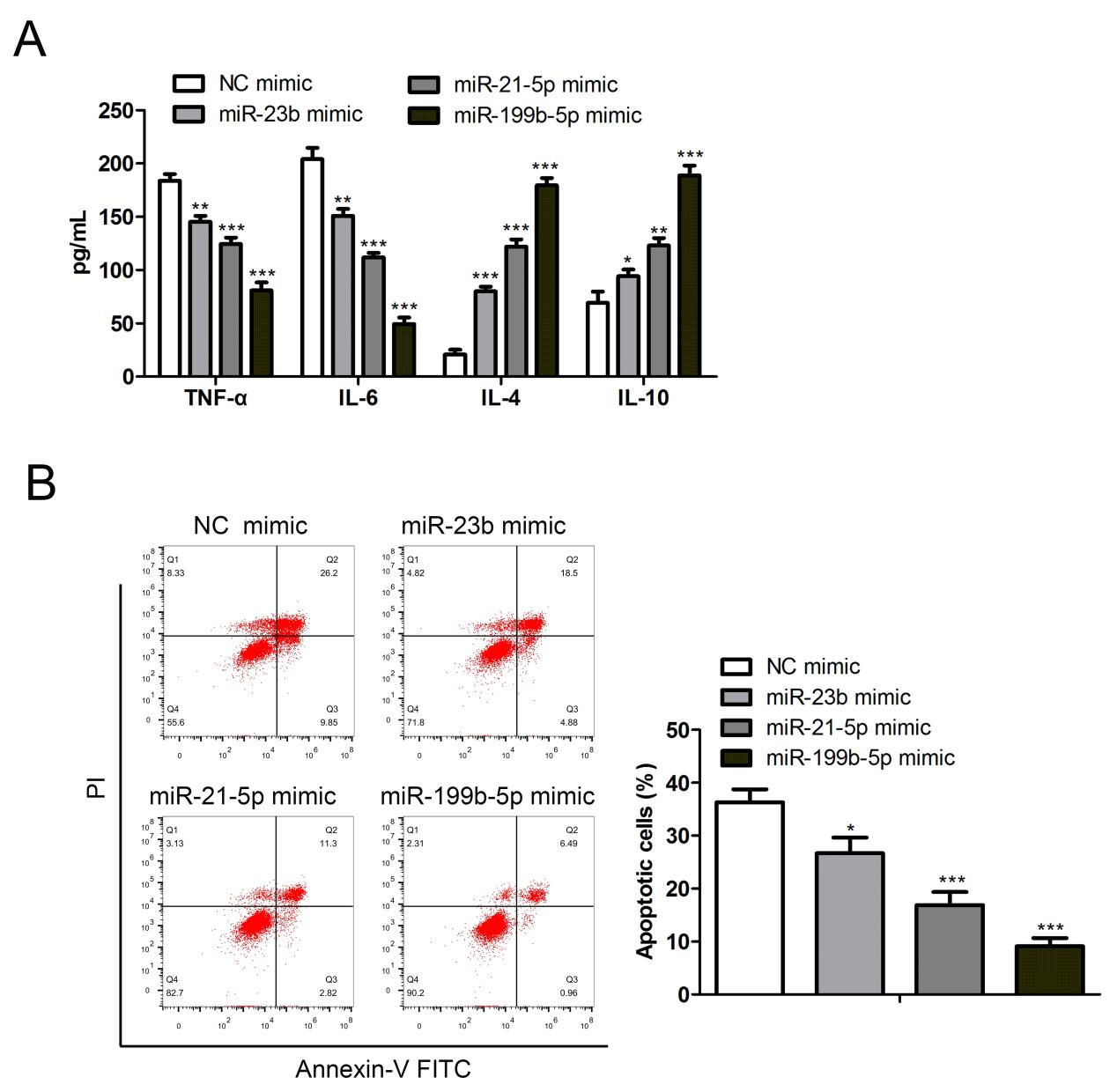


**Figure S1: MiR-23b，miR-21-5p and miR-199-5p inhibited neuroinflammation and reduced neuronal apoptosis.** (A) ElISA analysis detect inflammatory cytokines. (n = 3.) (B) The percentage of apoptotic neuronal cells was assessed using flow cytometry. (n = 3) **P <* 0.05, ***P <* 0.01, ****P <* 0.001.
